# Complete Genome Sequences of *Bacillus subtilis* subsp. *subtilis* Laboratory Strains JH642 (AG174) and AG1839

**DOI:** 10.1128/genomeA.00663-14

**Published:** 2014-07-03

**Authors:** Janet L. Smith, Jonathan M. Goldberg, Alan D. Grossman

**Affiliations:** a Department of Biology, MIT, Cambridge, Massachusetts, USA; b Broad Institute, Cambridge, Massachusetts, USA

## Abstract

The Gram-positive bacterium *Bacillus subtilis* is widely used for studies of cellular and molecular processes. We announce the complete genomic sequences of strain AG174, our stock of the commonly used strain JH642, and strain AG1839, a derivative that contains a mutation in the replication initiation gene *dnaB* and a linked Tn*917*.

## GENOME ANNOUNCEMENT

*Bacillus subtilis* is a soil bacterium studied in many laboratories and used commercially. It serves as a model for related Gram-positive pathogens. Many cellular processes are studied in *B. subtilis*, including transcription, translation, replication, metabolism, biofilm formation, sporulation, and horizontal gene transfer.

We report the genome sequence of *B. subtilis* subsp. *subtilis* strain AG174, our stock of strain JH642 (1). This widely used strain contains known point mutations conferring auxotrophy for tryptophan and phenylalanine and cold sensitivity (2, 3). We also report the sequence of AG1839 (also known as KPL69), a derivative of AG174 used in studies of replication initiation (4). It contains a mutation in the replication initiation gene *dnaB*, conferring a temperature-sensitive phenotype, and a linked Tn*917* insertion.

For AG174, 7.9 million 36-bp Illumina HiSeq reads and 0.88 million 150-bp paired-end reads were aligned to the AG1839 sequence (see below). The final circular sequence was 4,188,369 nucleotides (nt), with 98× mean coverage. Comparison of the finished AG174 genome to the previously reported JH642 genome (GenBank accession number CM000489.1 [3]) using Mauve (5) indicated that the genomes were 99.6% identical, with ~400 indels and ~30 point mutations. The vast majority of these differences appeared to be due to sequencing or alignment issues with the previously reported sequence. In addition, 269 unknown nucleotides (Ns) in the CM000489.1 sequence were resolved.

For AG1839, we used Bowtie (6) to align 15.8 million 40-bp reads to the previously sequenced version of JH642 (3). We used three rounds of alignment and refinement, followed by *de novo* assembly (7) to place the Tn*917* insertion in *ydbT*. PCR and conventional sequencing were used to determine the junctions of a 10.8-kb deletion that fuses *ppsC* and *ppsD* (encoding plipistatin synthetase). AG1839 differed from the parental AG174 by the expected point mutation in *dnaB* and the linked Tn*917*. Two additional point mutations were also present, in *thrS* (T85A) (4 kb upstream of *dnaB* linked to Tn*917*) and in *ytnA* (M105I) (60 kb downstream of *dnaB*). The final circular sequence was 4,193,640 nt, with 151× mean coverage.

Initial gene sets were predicted using Prodigal (8). The genome sequence of *B. subtilis* 168 (GenBank accession no. NC000964 [9, 10]) was aligned with those of AG174 and AG1839 using NUCMER (11). Coordinates from the alignments were used to add gene models missed by Prodigal and to map gene names and symbols to the new annotations. Gene models with potential problems were corrected or dropped if necessary, resulting in 4,227 and 4,231 predicted protein-coding genes for AG174 and AG1839, respectively. For both genomes, 86 tRNA genes were predicted using TRNASCAN (12) and 10 rRNA operons were predicted using RNAmmer (13).

AG174 differs from PY79, another commonly used laboratory strain (14). AG174 contains ICE*Bs1* and SP beta. There are 1,734 single-nucleotide polymorphisms (SNPs) in a 71-kb hypervariable region from *panB* to *hepT* and 35 SNPs distributed over the rest of the genome.

### Nucleotide sequence accession numbers.

The complete genome sequences are in GenBank, accession numbers CP007800 (AG174) and CP008698 (AG1839). AG174 and AG1839 are available from the Bacillus Genetic Stock Center.
